# Extracorporeal Organ Support for Burn-Injured Patients

**DOI:** 10.3390/ebj5020006

**Published:** 2024-03-25

**Authors:** Garrett W. Britton, Amanda R. Keith, Barret J. Halgas, Joshua M. Boster, Nicholas S. Niazi, Kevin K. Chung, Leopoldo C. Cancio

**Affiliations:** 1 School of Medicine, Uniformed Services University of the Health Sciences, Bethesda, MD 20814, USA; 2 US Army Institute of Surgical Research, Fort Sam Houston, TX 78234, USA; 3 Brooke Army Medical Center, Fort Sam Houston, TX 78234, USA; 4 David Grant US Air Force Medical Center, Travis Air Force Base, CA 94535, USA

**Keywords:** burn, carbon dioxide, extracorporeal, oxygenation, purification, kidney

## Abstract

As mortality relating to severe acute burn injury improves, patients are surviving longer into the critical care phase, which is commonly complicated by multisystem organ failure. Extracorporeal organ support (ECOS) represents a set of potential therapeutic technologies for managing patients with organ-specific complications. This article provides a comprehensive review of the existing literature, focusing on the use of continuous kidney replacement therapy, extracorporeal membrane oxygenation, extracorporeal carbon dioxide removal, and extracorporeal blood purification. Though promising, many of these technologies are in the early phases of implementation and are restricted to well-resourced medical systems, limiting their use in large scale casualty and austere scenarios.

## 1. Introduction

Burn injuries represent a devastating form of trauma, often leading to extensive tissue damage, fluid imbalances, and systemic inflammation. The management of severely-burn-injured patients poses significant challenges to healthcare providers, requiring a multidisciplinary and system-based approach to improve outcomes and reduce morbidity and mortality rates.

Over recent decades, a significant reduction in mortality relating to large burn injuries has been achieved, but as more patients are surviving to the later stages of critical illness and wound management, they experience increasing occurrences of sepsis and organ-related complications including renal failure and lung injury [1]. Sepsis and multisystem organ failure (MSOF) exceed burn shock as the leading causes of death in patients who survive the initial injury, necessitating the need for the early prediction and intervention for burn sepsis and novel organ-specific support [2].

In recent military conflicts, advancements in critical care medicine and en route care have paved the way for novel extracorporeal organ support strategies. Continuous kidney replacement therapy (CKRT), extracorporeal membrane oxygenation (ECMO), and extracorporeal blood purification (EBP) have emerged as promising avenues in the treatment of complications related to severe burn injury.

## 2. Kidney Replacement Therapy (KRT)

Severe burn injury is commonly associated with acute kidney injury; 20–46% of patients with burns of ≥20% total body surface area (TBSA) will develop some degree of renal dysfunction [3,4]. This reflects not only impaired renal perfusion during shock states, but also the effects of inflammatory mediators. Prior to the adoption of continuous therapy, burn patients with an indication for dialysis experienced a nearly 100% mortality rate [5,6]. CKRT has revolutionized the management of acute kidney injury (AKI) in critically ill patients. By providing continuous, slow, and precise removal of solutes and fluid, CKRT is better tolerated from a hemodynamic standpoint than conventional intermittent hemodialysis [7]. A recent subgroup analysis of a prospective multicenter study demonstrated a mortality benefit in burn patients in shock treated with continuous venovenous hemofiltration (CVVH) compared to all other modalities (54 vs. 37%; *p* = 0.032) [8].

### 2.1. Indications/Initiation

Absolute indications for initiating KRT in critically ill patients with AKI include refractory hyperkalemia, acidosis, volume overload, and clinical uremia. There is some debate regarding the optimal timing of KRT initiation in patients without these indications. Early initiation allows for improved control over metabolic parameters and fluid balance; delayed initiation (until indications develop) allows time for recovery and may obviate the need for KRT. There have been a number of trials in the general critical care population that have attempted to address this question. In earlier trials, there appeared to be a mortality benefit with early initiation, but more recent larger trials have failed to confirm this benefit [9]. In the largest trial, STARRT-AKI, over 3000 critically ill patients with KDIGO Stage 2 or 3 AKI without urgent indications for KRT were randomized to accelerated (within 12 h of randomization) vs. standard initiation of KRT (development of indication for KRT or persistent AKI > 72 h) [10]. There was no mortality difference between the accelerated and standard initiation arms (RR 1.00; 95% CI, 0.93 to 1.09; *p* = 0.92). Additionally, the accelerated arm was associated with higher rates of dialysis dependence and adverse events. However, burn patients were unrepresented in recent trials on KRT timing and differ from typical critically ill patients in terms of their catabolic state, high nutritional requirements, and complex volume status. There is limited evidence about the timing of KRT in patients with severe burn injuries; retrospective studies have shown an improvement in outcomes compared with historical controls with early initiation [11]. Overall, the optimal timing of KRT in burn patients is uncertain and further research is warranted.

### 2.2. CKRT Modality

A recent multicenter observation study demonstrated continuous venovenous hemofiltration (CVVH) was the most commonly prescribed CKRT modality compared to continuous venovenous hemodialysis (CVVHD) and continuous venovenous hemodiafiltration (CVVHDF) at 54%, 14%, and 30%, respectively [11]. The primary difference between CKRT modalities relates to the mechanism of solute clearance, namely convective (solute drag) vs. diffusive clearance. The authors hypothesize a benefit of convective clearance as a means to more efficiently clear larger molecular weight solutes, such as those mediators of inflammation and metabolic byproducts [12]. To date, there have been no studies demonstrating a definitive benefit of a single mode of renal replacement therapy.

### 2.3. CKRT Dosing

In the non-burn population, usual dosing for CKRT is considered to be an effluent flow rate of 25–30 mL/kg/h, to achieve a delivered dose of 20–25 mL/kg/h, allowing for periodic circuit exchanges and operating room visits [13]. In the burn population, usual dosing tends to be higher due to the postburn catabolic state; the average prescribed dose in a recent multicenter study was 42 mL/kg/h and the average delivered dose was 37 mL/kg/h [14]. The variation between the prescribed dose and the delivered dose is important for prescribing providers to note. Burn patients frequently experience interruptions in KRT because of issues such as daily wound care, surgical procedures, and catheter/circuit dysfunction, refer to Table 1.

The high-volume hemofiltration (HVHF) strategy is derived from the broader concept of blood purification, wherein hemofiltration is dosed to modulate the host response by removing the host and pathogen factors responsible for dysfunctional immune and inflammatory responses. CKRT doses > 40–45 mL/kg/h are typically considered to be in the range of HVHF [15]. Several studies have demonstrated a reduction in vasopressor dependency in patients with septic shock when treated with HVHF and, while no mortality benefit was observed, these studies were underpowered for this assessment [16,17,18,19]. It is important to note that this dosing exceeds the requirements of KRT alone and is intended to provide extracorporeal blood purification.

### 2.4. CKRT Anticoagulation

Hemofilter anticoagulation is utilized to prevent filter clotting, to extend filter life, and, thus, to limit interruptions in CKRT delivery. The two most commonly utilized strategies for anticoagulation are heparin and regional citrate. If heparin is used, it is typically infused pre-filter at a rate of 500 u/h. Some centers titrate heparin dose to target a PTT of 45–60 s, lower than therapeutic anticoagulation. If citrate is used, it is infused pre-filter and calcium must be infused post-filter to reverse its effects. There is significant practice variation in CKRT anticoagulation practices. A multicenter review of 170 patients receiving CKRT reported heparin use in 44% and citrate use in 52% of cases. Interestingly, 53% of CKRT cases were managed without regional or systemic anticoagulation [14]. A recent parallel multicenter study (n = 638) comparing systemic heparin to regional citrate demonstrated a prolonged filter life in patients managed with regional citrate: 47 h vs. 26 h (*p* < 0.001) [19]. Due to the need for additional monitoring and the potential toxicity related to citrate administration, as well the ease of heparin administration, regional heparin is commonly utilized. Lastly, in the presence of an indication for systemic anticoagulation such as pulmonary embolism, deep vein thrombosis, or acute myocardial infarction, regional anticoagulation is not required in addition to systemic therapy.

### 2.5. Discontinuation of Therapy

Liberation from KRT poses a challenge in burn patients because of persistent hypermetabolism, deranged volume states, and the recurrent risk for renal injury on the backdrop of ill-defined criteria for the discontinuation of therapy. Current guidelines recommend the discontinuation of therapy “when RRT is no longer required either because intrinsic kidney function has recovered to the point that it is adequate to meet patient needs, or because RRT is no longer consistent with the goals of care” [20]. A recent multicenter study demonstrated key characteristics associated with the successful discontinuation of CKRT, which included higher 24 h urine output following KRT discontinuation (2.4 L vs. 1.6 L; *p* = 0.005), higher creatinine clearance (29 mL/min vs. 7 mL/min; *p* < 0.001), and lower creatinine ratio (day 2/day 0 after KRT discontinuation, 1.16 vs. 1.63; *p* < 0.001) [21].

AKI in this population does not appear to confer a high risk of the long-term need for KRT when treated with CKRT, similar to the general critically ill patient population. A multicenter study indicated only 21% of burn patients required KRT at the time of discharge and only 6% required KRT beyond six months following hospital discharge [14]. For comparison, in the STAART AKI trial, the rate of KRT dependence at 90 days in the patients enrolled in the standard KRT initiation arm was also 6% [10].

## 3. Extracorporeal Membrane Oxygenation (ECMO)

Acute respiratory distress syndrome (ARDS) is common in severe burn patients who require mechanical ventilation, with incidence rates estimated to be between 20% and 60% [22,23]. Burn-related ARDS may arise from a variety of factors, including smoke inhalation, fluid resuscitation, ventilator-associated pneumonia, or systemic inflammation resulting from the burn injury itself. The mortality rate of burn-related severe ARDS has been reported to be as high as 50%, compared to non-burn severe ARDS of 40% [23]. The conventional management of ARDS includes lung-protective ventilation with low tidal volumes, fluid restriction, and prone positioning. When conventional treatment strategies fail, extracorporeal membrane oxygenation (ECMO) may be an option.

ECMO acts as a life-sustaining support by providing extracorporeal oxygenation and carbon dioxide removal, thereby alleviating the stress on damaged lungs and facilitating lung recovery. By offering an extended window for lung healing, ECMO has the potential to improve gas exchange and reduce ventilator-associated complications, ultimately enhancing the survival chances of severely burned individuals.

ECMO is rapidly gaining popularity for the management of severe ARDS in the general intensive care population, and the Extracorporeal Life Support Organization (ELSO) has reported survival rates ranging from 40% to 60% [24]. The specific indications for ECMO in burn patients remain relatively unclear and require further investigation. Most of the existing literature consists of case studies, case reports, and case–control studies which have reported inconsistent mortality rates ranging from 43 to 91% [22,23,25,26,27]. Asmussen et al. in 2013 published the first meta-analysis of ECMO in burn patients and suggested that ECMO did not improve survival in burn patients with ARDS [28]. A meta-analysis published in 2022 reported that burn patients with inhalation and revised Baux scores exceeding 90 had lower-than-predicted mortality when treated with ECMO [29]. Most recently, a meta-analysis of 15 retrospective studies including 318 burn patients with severe ARDS reported successfully weaning from ECMO in 67% of patients and hospital survival in 51% of patients; this is consistent with ECMO survival in the general intensive care population reported by the ELSO [30]. The majority of studies included in the meta-analyses mentioned above lacked control groups, which makes it difficult to make strong conclusions regarding the benefit of ECMO in the burn population. Our conclusion is that referring providers should not be deterred from offering ECMO in burn-injured patients based on usual indications.

Despite the lack of high-quality evidence, ECMO use in North American burn centers is becoming increasingly common. A recent survey reported that nearly half of the centers utilized ECMO for the management of burn-related ARDS [31]. The risk of complications is higher in the burn population than in the general ICU population (67.5% vs. 40.2%, respectively) [32]. These include infection, bleeding, and thrombosis among others. Infection rates have been reported to be three times more common in burn patients requiring ECMO than in non-burn ECMO patients [33]. This may be attributable, in part, to a delay in definitive surgical burn wound closure, occasioned by the inability to operate due to the use of systemic anticoagulation to prevent the thromboembolic complications associated with ECMO. The optimal management of systemic anticoagulation in burn ECMO is unclear and reports of ECMO run entirely without anticoagulation are being reported with increasing frequency in the non-burn ECMO population [34,35]. We suspect that given the high bleeding risk and frequent need for invasive procedures in the burn population, the ability to safely interrupt systemic anticoagulation in burn ECMO would improve outcomes. We are optimistic that advances in ECMO circuitry may minimize the requirement for systemic anticoagulation in the future, although current international guidelines endorsed by the ELSO recommend systemic anticoagulation, unless contraindicated due to the risk for circuit thrombosis, which could result in catastrophic complications or death [34,35]. Thus, the decision to place a patient on ECMO must be individualized and made in concert with a multidisciplinary team (refer to Table 2). Wound status should be considered, as follows: unexcised; excised, grafted, but not healed; or excised, grafted, and healed.

## 4. Extracorporeal Carbon Dioxide Removal

Extracorporeal carbon dioxide removal (ECCO_2_R) has recently emerged as a promising therapy in the management of patients with respiratory failure. Traditional mechanical ventilation may cause ventilator-induced lung injury (VILI), in turn necessitating the use of escalating tidal volumes and pressures to maintain adequate oxygenation and ventilation [36,37,38]. ECCO_2_R involves a similar technology but uses smaller cannulas and lower blood flow rates than full ECMO. The goal is to perform a significant portion of carbon dioxide removal while providing some oxygenation as well. Thus, ECCO_2_R should permit lower peak inspiratory pressures and minute ventilation, reduce the risk of ventilator-induced lung injury (VILI), and permit earlier weaning from mechanical ventilation (refer to Table 3) [39,40].

Despite the potential advantages of ECCO_2_R, several challenges merit attention. First, the selection of appropriate candidates for ECCO_2_R remains crucial. Additionally, there is a need for further research to elucidate the ideal timing, duration, and settings of ECCO_2_R in burn patients, as well as its long-term impact on patient outcomes and quality of life. Circuit-related issues, bleeding, infection, and thrombosis are among the known adverse events associated with ECCO_2_R; monitoring and expertise in the management of these complications are essential. Research on ECCO_2_R in burn patients is ongoing.

## 5. Extracorporeal Blood Purification

The pathophysiology of multisystem organ failure following extensive burns (≥20% TBSA) includes a pronounced, unbalanced, and sustained inflammatory response to injury. This systemic reaction is similar to other dysregulated inflammatory responses such as pancreatitis, autoimmune disorders, trauma, and sepsis. Thus, exploratory treatment modalities for burn shock have been extrapolated from those illnesses.

Extracorporeal blood purification (EBP) is gaining traction within the sepsis and burn community to help mitigate this dysregulated inflammatory response [41]. EBP methods can be classified by where in the inflammatory pathway they act, as follows: removal of pathogens; adsorption of endotoxin or cytokines; or modulation of activated neutrophils.

### 5.1. Total Plasma Exchange (TPE)

Plasma exchange, or plasmapheresis, has been used in a few centers for the treatment of refractory burn shock. TPE replaces plasma with either albumin or fresh frozen plasma in order to remove cytokines and other pro-inflammatory mediators [42]. Use of TPE in burn patients was derived from its use in diseases such as Guillain Barre syndrome, myasthenia gravis, sepsis, and others [43].

Studies exploring TPE in burn shock have had mixed results [42,44,45]. In 1983, Warden et al. published a retrospective review of 22 patients wherein TPE was found to reduce the total fluid requirements and lactate levels during resuscitation [46]. The first randomized control trial (RCT) by Kravitz et al. had 17 patients. They found that TPE did not alter the course of burn shock or fluid requirements [45]. However, TBSA was higher in the treatment group, introducing bias into the results. A study by Ninnemann et al. showed that TPE aided in restoring lymphocyte immune function [44]. More recently, a retrospective analysis by Neff et al. in 2009 examined 21 patients with greater than 20% TBSA who underwent TPE if they received high resuscitation volumes, defined as being 1.2-fold greater than the amount predicted [47]. There was a significant reduction in lactate levels and intravenous fluid infusion rates, as well as an increase in the mean arterial pressure and urine output. No large RCT has been carried out to support routine TPE use in burn care.

### 5.2. Pathogen Removal

Sepsis is the leading cause of mortality (51%) in burn patients [2]. There is growing evidence to support methods of pathogen removal through selective filters. The Seraph 100 filter (ExThera Medical, Martinez, CA, USA), for example, uses polyethylene beads coated with heparin to mimic the heparan sulfate present in the epithelial glycocalyx. The heparin-coated beads can bind to bacteria, fungi, and viruses, removing them from the blood [38]. This modality was utilized for the treatment of COVID-19 in 2020, with favorable results. A retrospective observational study by Chitty et al. showed a mortality reduction from 64% to 32% in 53 patients with severe COVID-19 [48]. There are both active and planned RCTs in Europe and the US to evaluate the Seraph 100 in bacteremic patients, including burn patients.

Other pathogen-removal methods that are currently under investigation are the GARNET hemofilter and the Hemopurifier. The GARNET hemofilter (Boa Biomedical, Inc., Cambridge, MA, USA) uses mannose-binding lectin on the filter as an opsonin to bind pathogens [41,49]. It has also been found to remove pathogen-associated molecular patterns (PAMPs) from the circulation in septic rats [47]. The Hemopurifier (Aethlon Medical, Melbourne, Australia) uses plasmapheresis and lectin proteins within the filter to remove viruses and virus-infected cells [41,50,51]. Both modalities are currently enrolling, utilizing the GARNET hemofilter in bacteremia and the Hemopurifier for treatment of severe COVID-19.

### 5.3. Cell-Directed Extracorporeal Therapy

The Selective Cytopheretic Device (SCD) (SeaStar Medical, Denver, CO, USA), is a technology that sequesters and modulates the most activated neutrophils and monocytes [52]. The SCD is a biocompatible membrane that mimics capillary blood flow and sequesters activated leukocytes [53]. The SCD is utilized in conjunction with CKRT, with the SCD downstream from the CKRT hemofilter in the circuit. The SCD requires the use of regional citrate anticoagulation to induce hypocalcemia within the SCD, which results in both the inhibition of the clotting cascade but also the inactivation of leukocytes. There are a number of smaller studies in critically ill medical patients which have shown promising results, but there are limited data including burn patients [54,55,56,57]. A multicenter randomized controlled trial, NEUTRALIZE-AKI, is currently enrolling critically ill patients with AKI, who require CKRT [58].

### 5.4. Endotoxin, Cytokine, and PAMP Adsorption

Adsorption columns aim to bind endotoxins, cytokines, and other pro-inflammatory mediators. The columns are largely nonselective, working in conjunction with CKRT or as stand-alone systems. Some of the membranes under investigation are polymethyl-methacrylate (PMMA) membranes, oXiris, CytoSorb, and the polymyxin-B endotoxin-adsorbing column [59].

One method of endotoxin elimination is Polymyxcin-B (PMX), an antibiotic embedded into the hemoperfusion cartridge that binds endotoxin with high efficiency [41,60]. In a small study in burn patients, the PMX filter was found to significantly decrease endotoxin, IL-1 beta, IL-6, and IL-8 levels in those receiving CKRT [61]. In contrast, the EUPHRATES trial, an RCT using PMX-embedded cartridges in non-burn septic patients, did not show a reduction in mortality at 28 days [60]. While PMX hemoperfusion reduces inflammatory mediators, the clinical benefit of using PMX is still uncertain.

The poly(methyl methacrylate) (PMMA) membrane has a hydrophobic, symmetric, microporous structure that enables it to adsorb cytokines, beta-2 microglobin, and immunoglobins [58]. There has been a case series on the use of PMMA, but no large RCTs, to assess its utility in burn patients [40]. Results from a cohort study by Nakada et al. using PMMA in septic patients showed a reduction in IL-6 and lactate levels and in vasopressor requirements [62].

The CytoSorb filter (CytoSorbents, Monmouth Junction, NJ, USA) is composed of a porous polymer of adsorbent beads that bind molecules via hydrophobic interactions up to a size of 60 kDa. The binding is non-selective and concentration dependent, such that substances at higher concentrations are adsorbed more readily. In vitro studies report that CytoSorb decreases cytokines such as TNF-α, IL-10, IL-6, and IL-1β by more than 90%, but not levels of endotoxin [63,64]. Brouwer et al. published a retrospective study involving 67 patients who received CKRT with CytoSorb and 49 patients with CKRT alone in the setting of septic shock. The CytoSorb patients had a lower overall mortality, 53% vs. 72% [65]. There are ongoing studies investigating CytoSorb in various inflammatory conditions. One clinical trial (NCT04195126) is studying the use of CytoSorb in burn patients with greater than 20% TBSA.

Although the current focus for these EBP technologies is primarily on the treatment of sepsis, there may be a possible role for the management of the extensive inflammatory response to burns and other traumatic injury. Multiple studies have demonstrated severe and dysregulated inflammatory responses to burns and other traumatic injuries, mimicking that of sepsis, and associated with commensurate mortality rates [66,67,68]. The authors hypothesize a benefit of attenuating a severe inflammatory response in acute burn injury, as it relates to wound healing, modulating hypermetabolic response and limiting organ failure.

## 6. Military Relevance

During recent conflicts in Iraq and Afghanistan, US forces relied on a well-coordinated system that delivers care through increasingly more resourced echelons (“roles”) of care, with the goal of quickly removing injured warfighters from the battlefield. This process depends on access to rapid aeromedical evacuation, which is only achieved in the setting of air superiority. Under these circumstances, there is little incentive to position extracorporeal resources far forward on the battlefield.

On the other hand, the military has already established that extracorporeal technology can be effectively used during aeromedical evacuation. Operated jointly by the U.S. Air Force’s 59th Medical Wing (MDW), Brooke Army Medical Center, and the U.S. Army Burn Center’ Burn Flight Team, the U.S. military ECMO program transported 110 patients between 2012 and 2019 [69]. The majority of these patients were cannulated by the team prior to transport. The most common indication was acute respiratory failure. Because of the inherent complexities of extracorporeal therapies, resources and expertise are naturally consolidated to high-volume centers. Air-based transport teams help to make the technology more accessible worldwide.

CKRT has similarly been utilized in forward operating environments and evacuations which have demonstrated favorable results [70,71,72]. In 2010, CKRT was incorporated into a Role 3 facility in Afghanistan and provided care to nine service members over the course of 14 months [71]. The majority of the patients were blast victims who quickly developed critical hyperkalemia. Renal replacement allowed for the immediate stabilization of the patients prior to transport to a higher level of care. Mean duration of pre-flight therapy was 18 h. Therapy was interrupted for transport but was resumed on arrival in Germany. The authors attributed the success of the program to the location of the hospital (regional air hub), a “physician champion”, and nursing staff with prior CKRT experience. One of the challenges they identified was the rapid turnover of hospital personnel. To that point, the longevity of such a program relies heavily on leadership buy-in, with an emphasis on assigning qualified personnel to that location. Driscoll et al. describe the first recorded in-flight use of CKRT for intercontinental aeromedical evacuation of a severely burned U.S. service member. A physical challenge was that the system (NxStage) had to conform to flight-approved container weights, which the authors overcame by disassembling the screen, processor, and fluid warmer prior to use. The patient received 15 h of therapy within 19 h of travel time. The circuit was interrupted only for ascent, descent, and aircraft changes. Even though the patient received systemic anticoagulation, the filters were routinely exchanged during these scheduled breaks to further avoid untimely thrombotic events [72].

U.S. planners anticipate that the next major conflict will likely involve a near-peer adversary and large-scale combat operations. If so, air superiority will not be assured, aeromedical evacuation will be delayed, and casualties will require prolonged field care at lower echelons under austere conditions. Patients are, thus, more likely to develop infections and organ failure before arriving at definitive care (Role IV) hospitals. We anticipate that this will increase the need for extracorporeal technologies, such as those described in this article, to support casualties coming off the battlefield with ARDS, AKI, and/or sepsis. To fill this need, there will likely be efforts to miniaturize and simplify the technologies to enhance their utility in more remote settings.

## 7. Conclusions

The introduction of extracorporeal organ support into the management of burn patients during recent military conflicts represents an emerging field of novel technologies with potential benefit in critical care medicine. The implementation of continuous kidney replacement therapy, extracorporeal membrane oxygenation, and extracorporeal blood purification in burn patients has demonstrated promising results in mitigating organ dysfunction, enhancing metabolic balance, and ameliorating systemic inflammation. Extending such extracorporeal capabilities to severely injured individuals as close to the point of injury as possible may ameliorate the challenges associated with future large-scale combat operations and other challenging burn-care scenarios.

## Figures and Tables

**Table 1:** CKRT therapy fluid dosing (based on effluent flow).

Usual Dosing	Dosing in Burns	High Volume Hemofiltration
25–30 mL/kg/h	30–40 mL/kg/h	>45 mL/kg/h

**Table 2:** Indications for ECMO Consultation.

Hypoxemic respiratory failure with PaO_2_/FiO_2_ < 80 mm Hg, after optimal medical management.
Hypercapnic respiratory failure with pH < 7.25, despite optimal conventional mechanical ventilation.
Ventilatory support as a bridge to lung transplantation or primary graft dysfunction following lung transplant

**Table 3:** Comparison of technical characteristics (ECCO_2_R vs. ECMO).

Parameter	ECCO_2_R	VV-ECMO
Typical blood flow rate	250–450 mL/min	1000 mL/min–5000 mL/min
Typical cannula size	One dual-lumen cannula (13–23 Fr)	Two single-lumen cannulas (21–29 Fr) or one dual-lumen cannula (27–31 Fr)
Anticoagulation required	Regional/systemic	Systemic
